# X-ray spectroscopic study of amorphous and polycrystalline PbO films, α-PbO, and β-PbO for direct conversion imaging

**DOI:** 10.1038/s41598-017-13703-7

**Published:** 2017-10-13

**Authors:** A. Qamar, K. LeBlanc, O. Semeniuk, A. Reznik, J. Lin, Y. Pan, A. Moewes

**Affiliations:** 1 Department of Physics and Engineering Physics, University of Saskatchewan, 116 Science Place, Saskatoon, SK S7N 5E2 Canada; 2 Chemistry and materials science program, Lakehead University, 955 Oliver Road, Thunder Bay, ON P7B 5E1 Canada; 3 Advanced detection devices department, Thunder Bay Regional Health Research Institute, 290 Munro Street, Thunder Bay, ON P7A 7T1 Canada; 4 Department of Physics, Lakehead University, 955 Oliver Road, Thunder Bay, ON P7B 5E1 Canada; 5 The Department of Geological Sciences, 114 Science Place, University of Saskatchewan, Saskatoon, SK S7N 5E2 Canada

## Abstract

We investigated the electronic structure of Lead Oxide (PbO) – one of the most promising photoconductor materials for direct conversion x-ray imaging detectors, using soft x-ray emission and absorption spectroscopy. Two structural configurations of thin PbO layers, namely the polycrystalline and the amorphous phase, were studied, and compared to the properties of powdered α-PbO and β-PbO samples. In addition, we performed calculations within the framework of density functional theory and found an excellent agreement between the calculated and the measured absorption and emission spectra, which indicates high accuracy of our structural models. Our work provides strong evidence that the electronic structure of PbO layers, specifically the width of the band gap and the presence of additional interband and intraband states in both conduction and valence band, depend on the deposition conditions. We tested several model structures using DFT simulations to understand what the origin of these states is. The presence of O vacancies is the most plausible explanation for these additional electronic states. Several other plausible models were ruled out including interstitial O, dislocated O and the presence of significant lattice stress in PbO.

## Introduction

There is growing research interest in the wide band gap photoconductors used as x-ray-to-charge transducers in direct conversion medical imaging detectors^1,2^. In direct conversion detectors, a uniform layer of photoconductor material deposited on imaging electronics is used to convert the absorbed x-rays directly to collectable charge carriers (electron-hole pairs). Since x-ray image detectors always need to cover a large area, the use of a single crystalline photoconductor in direct conversion detectors is not feasible. Thus, one has to concentrate on polycrystalline or amorphous modifications of higher atomic number materials deposited directly on the imaging array. Potential polycrystalline x-ray photoconductor materials compatible with large area detector technology include PbI_2_, HgI_2_, TlBr, Zn_x_Cd_1−x_Te, and PbO. Despite intensive research none of these materials are currently optimized for commercial use. The major obstacles include their poor transport properties and low carrier ranges governed by the mobility-lifetime product, inherent to polycrystalline materials and due to the charge carrier trapping at the grain boundaries. Indeed, if drifting x-ray-generated carriers are captured into deep traps from which there is no escape over the time scale of interest, the charge collection efficiency is reduced resulting in loss of detector sensitivity^3–6^ and image blur^7,8^. Currently, only amorphous Selenium (a-Se) is being used commercially in direct conversion detectors although its x-ray detection quantum efficiency is not large enough. The major advantage of a-Se over polycrystalline materials in the development stage listed above is that both types of carriers are mobile in this material at practical electric fields. The disadvantage of a-Se is its comparatively low Z (atomic number) that makes it suitable only for applications with soft x-rays such as mammography. For a wider diagnostic energy range, i.e. for general radiographic and fluoroscopic applications, a-Se has to be replaced with a higher Z material. PbO holds a special place in the above list of potential photoconductors because, like a-Se, it has a long and successful history of utilization in optical imaging: thin layers of polycrystalline PbO are employed in so-called Plumbicon video pick-up tubes extensively used both for broadcast and for fluoroscopy and digital subtraction angiography in conjunction with image intensifiers. However, the technological transfer from thin films used in optical imaging to thick layers needed for x-ray imaging is less successful: the first prototype of a PbO flat panel detector for radiography showed a very high spatial resolution but suffered from incomplete charge collection and *image lag*, which is caused by the presence of residual current after the exposure^1,8–11^. This is not surprising considering its polycrystalline structure. To overcome these problems, we have recently proposed to modify the PbO deposition method to achieve an amorphous (or glassy) PbO structure and to eliminate grain boundaries^12^.

Here, we report on the electronic structure of amorphous PbO studied with synchrotron-based soft x-ray emission (XES) and absorption spectroscopy (XAS) and compare structures of differently synthesized PbO samples including the conventional polycrystalline PbO. XAS in conjunction with XES is a powerful experimental technique which directly probe the valence and conduction bands of a material. Surprisingly, despite its extensive commercial use, relatively little is known about the electronic structure of various lead oxides. Even though a few previous studies have investigated the electronic structure, such as density of states and band diagrams using first principle calculations^13–17^, a very limited amount of experimental characterization has been reported on this class of materials. To the best of our knowledge there has been only one XAS and XES study on α-PbO^14^. No XAS and XES work has been published on either β-PbO or PbO films deposited under conditions used in flat panel detector technology. To better interpret our experimental results, we also performed DFT calculations. Our work shows that the presence of O vacancies in the amorphous sample introduces additional electronic states near the bottom of the conduction band and within the valence band. These states can be eliminated by thermal annealing.

## Results and Discussion

### XAS and XES: Assigning main peaks

**Figure 1:**
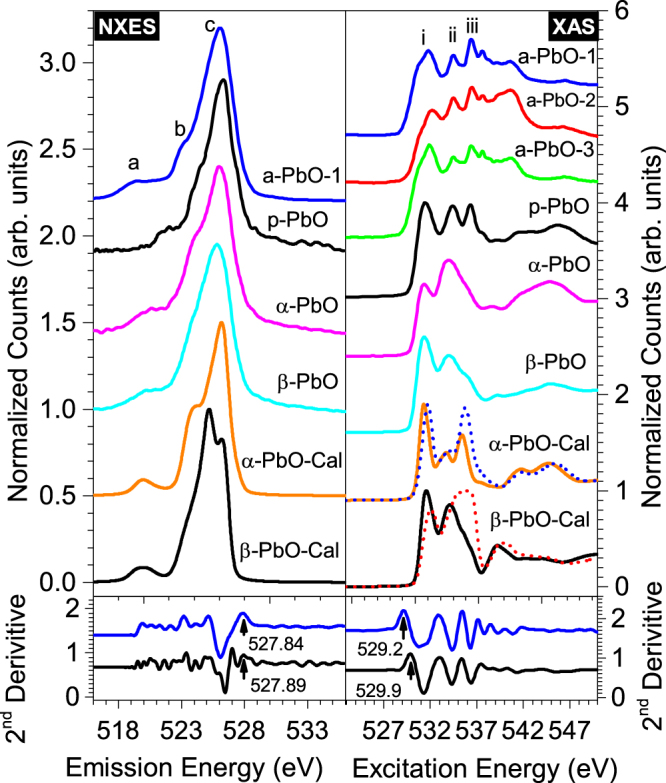
Top left panel: Calculated (Cal) and experimental non-resonant x-ray emission spectra (NXES). Top right panel: Calculated and experimental XAS. The dotted lines represent ground state calculated XAS, and solid lines represent XAS calculated with a supercell in which an O 1 s core hole is present reflecting the final state of the absorption process. The bottom panels show the 2^nd^ derivatives of the respective experimental spectra in order to determine the band edges.

We will first discuss the experimental and calculated XAS spectra for all the PbO samples shown in Fig. 1 on the right. For more detailed description of how these spectra were calculated, please refer to the subsections ‘DFT calculations’, and ‘Crystal structures’ in the section ‘methods and materials’. The top three spectra shown in this panel correspond to the three amorphous samples; a-PbO-1, a-PbO-2, and a-PbO-3. Note that when we mention a-PbO we are referring to any of the three amorphous samples. There are three main features (i, ii, iii) for all the amorphous PbO (a-PbO) and polycrystalline PbO (p-PbO) samples. All these three features have O 2p and Pb 6 s character. These bands also appear for measured XAS for both powdered α-PbO and β-PbO samples, but the relative intensity of peak iii is strongly suppressed in both powdered samples. They are all present in the calculated XAS for both α-PbO and β-PbO. However, the relative intensity of peak ii is underestimated in the calculated XAS spectrum for the alpha phase, while the relative intensity of peak iii is underestimated in the calculated XAS spectrum for β-PbO. For all the calculated XAS spectra with an O 1 s core hole present, the main features are shifted to the left by about 0.5 eV, but the onset in absorption is shifted by less than 0.05 eV.

The experimental and measured XES spectra are shown in Fig. 1 on the left. Three features (a, b, c) appear for all PbO samples. All of these three bands have O 2p character to a varying degree because O K-edge x-ray spectroscopy probes the localized density of states of p-symmetry at the O sites. The most intense feature labeled’c’ is just below the Fermi level, and it has the strongest O:2p character. Band ‘b’ is a hybrid of Pb:6p and O:2p. The peak labeled ‘a’ in all the PbO samples is very weak. It has Pb:6 s and O:2p hybridization. This is in disagreement with the traditional view that in PbO, Pb 6 s^2^ electrons behave as a chemically inert lone pair. The mixing of Pb:6 s with O:2p states supported by our DFT calculations and emission spectra clearly shows that these 6 s electrons involve in bonding with O atoms and hence are not chemically inert. This finding is in agreement with other studies based on DFT calculations^16,18,19^. Another study has suggested the presence of fine structure in Pb L edge due to tunneling of Pb:6 s^2^ electrons^20^. At our beamlines, we did not have access to high enough energy photons to probe the Pb L edge.

### Additional states at the bottom of CB for a-PbO samples

**Figure 2:**
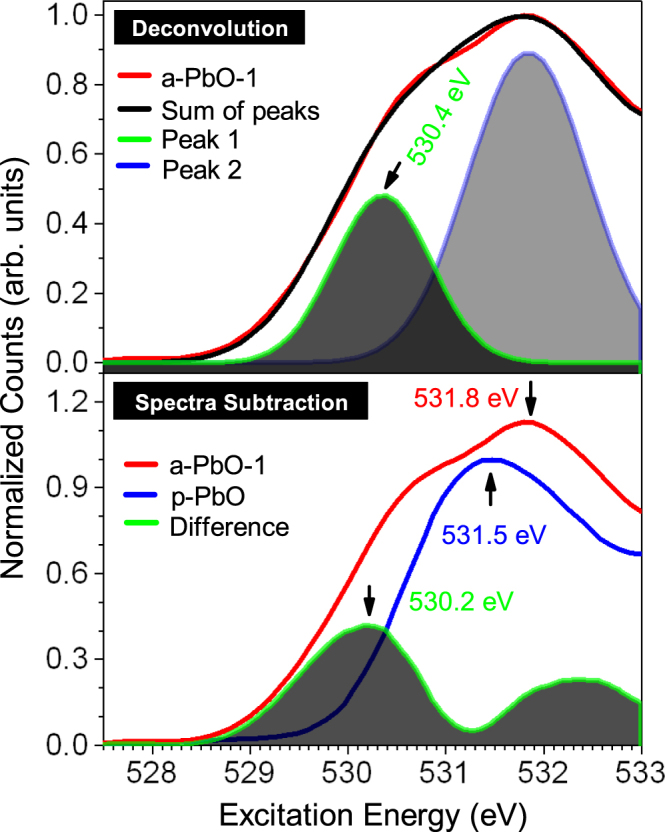
Top panel: Peak deconvolution analysis performed on experimental XAS for a-PbO-1 shows presence of additional localized DOS (green). Bottom panel: XAS spectra for a-PbO-1, p-PbO, and their difference near the CB edge (green).

We now turn to the discussion of a band tailing effect present in the electronic structure of a-PbO samples. The density of states near the bottom of the CB for all a-PbO tails off more gradually into the band gap instead of the relatively sharp decrease for all other PbO samples. Subsequently we find a ~0.7 eV lower onset in absorption for all the amorphous PbO samples (a-PbO) than for the p-PbO and powdered PbO samples. This is consistent with the recently proposed picture of polycrystalline PbO as a network of very thin (micrometer-size) platelets each of which is single crystalline^13^. Figure 2 illustrates the presence of these additional states for the a-PbO-1 sample. Even though we show only a-PbO-1 in this Figure, the tailing effect is seen in all amorphous samples.

Two different methods are used to extract more detailed information about how these states are distributed. The first method involves subtracting the a-PbO-1 XAS from the p-PbO XAS (bottom graph in Fig. 2). This allows determining how the band edge for the amorphous sample, a-PbO-1, differs from that of the polycrystalline sample shedding light on the distribution of these additional states. The result shows a Gaussian distribution with the presence of both interband and intraband states in the CB. Moreover, the band edge for the p-PbO sample is at 529.3 eV while the peak in this Gaussian distribution is located at 530.2 eV indicating that the additional DOS peak is located inside the CB (intraband) for the a-PbO-1 sample.

Similar information is obtained performing a peak deconvolution analysis on the measured XAS spectrum for a-PbO-1. This is shown in the top panel of Fig. 2. In this figure, the blue and green curves added together give the black curve, which agrees very well with measured XAS for a-PbO-1 shown in red color in Fig. 2. This method again shows a Gaussian distribution with the maximum in DOS located at 530.1 eV, and presence of both inter- and intraband additional states. Therefore, both of these analyses point to the same conclusion. This broadening of band edges is a general property of amorphous materials^21^. This effect results from the lack of long-range order in the amorphous structure and consists of spatially separated and energetically distributed additional states^22^. In an amorphous solid, the electrostatic potential, caused by the coulomb forces of individual nuclei, varies randomly in space. This disorder in the potential originates due the absence of long-range order in nuclei coordinates resulting in lack of lattice-like structure for such materials. As a result, the sharp band edges are smeared out resulting in electronic states within the otherwise energy forbidden gap. Thus both measured spectra and peak deconvolution analysis show the presence of an additional band near the bottom of CB. We will look more closely at what mechanism could possibly explain this but for now we conclude that as a result of these two effects, the XAS onset is shifted to a lower energy for the amorphous samples.

### Additional states inside the VB of a-PbO samples

**Figure 3:**
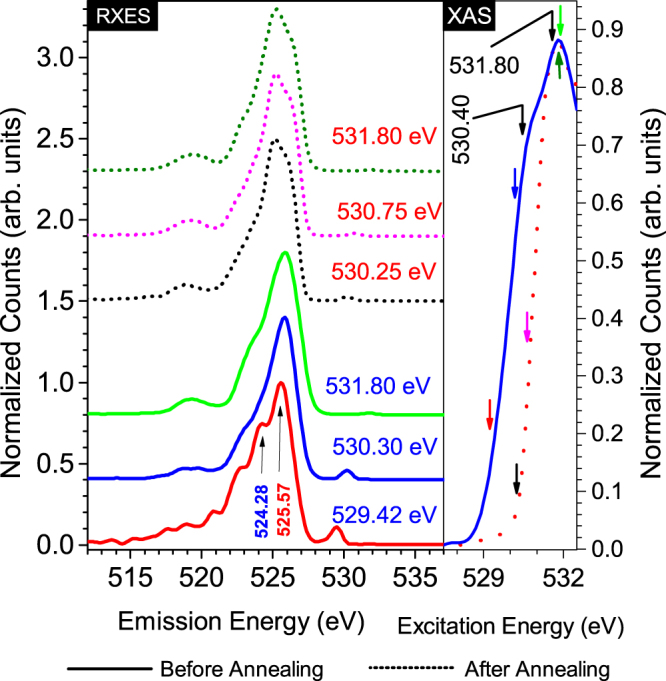
Left panel: Experimental resonant XES (RXES) at the indicated excitation energies indicating additional features in VB disappear after annealing. The solid lines represent spectra for a-PbO-1 before annealing, while the dotted lines represent spectra for a-PbO-1 after annealing. Right panel: Experimental XAS indicating the lack of additional states in CB after annealing. The smaller colored arrows indicate where along the absorption edge the sample is excited to obtain the emission spectra in the right panel with the matching color.

We will now turn to the discussion of how the valence bands for a-PbO-1 and p-PbO differ. In order to probe the valence bands more thoroughly, we turn to Resonant X-ray Emission Spectroscopy measurements (RXES). This method involves resonant excitation of a core electron (here O 1 s) to the conduction band coupled with the relaxation of a valence band electron to the core level and subsequent emission of a photon. Changes in the emission spectra are observed as the excitation energy is changed along the absorption edge. The RXES measurements for a-PbO-1 are shown in Fig. 3. The right panel of this Figure shows the excitation energies chosen along the absorption edge to obtain the corresponding emission spectra excited at that respective energy displayed in the left panel. When exciting at energies of 529.42 eV or larger, the XES spectra look exactly like all the other PbO samples we investigated (shown in Fig. 1). However, two new bands on the left of the main peak are clearly seen when exciting the a-PbO-1 sample at 529.42 eV or below. We submit that these new features are due to emission from additional states present only in the amorphous film samples. Our claim is further supported by our DFT calculations on model structures discussed below. These additional states lie well within the band. Furthermore, the top of the valence band shows very little or no broadening effect for the amorphous sample. Thus the valence band edge is not shifted in energy unlike the conduction band edge. RXES measurements performed on the p-PbO sample do not exhibit any appreciable difference in the emission spectra when exciting resonantly anywhere along the absorption edge.

### The effect of thermal annealing

Thermal annealing is a process that allows atoms in a crystal lattice to relax and therefore slightly rearrange thus reducing the long-range disorder. How effectively can thermal annealing reduce the disorder in our a-PbO samples? In order to answer this question, the a-PbO-1 sample is thermally annealed as discussed in the samples section. We perform RXES measurements on the annealed a-PbO-1 sample and observe how the results differ from the RXES measurements taken before annealing. The results are shown in Fig. 3. The right-side panel in this figure shows the absorption edge before and after the thermal annealing. The band edge becomes sharper and the additional band originally present on the left side of the main absorption feature is totally eliminated. The absorption spectrum looks very similar to p-PbO. The right-side panel of Fig. 3 shows RXES spectra collected at various excitation energies along the absorption edge. There is no noticeable difference in the emission spectra as the excitation energy is lowered all the way to the very bottom of the CB edge. Therefore, the additional features in the valence band also disappear due to thermal annealing. We conclude that thermal annealing recrystallizes the amorphous samples to the extent that their electronic structure becomes very similar to the polycrystalline sample.

### The model calculations

So far, we have only focused on the experimental evidence indicating the presence of the additional electronic states, but we would also like to know the physical explanation behind it. We first briefly discuss how amorphous materials differ from crystalline materials. In perfect crystalline samples, the nuclei are arranged in a perfect periodic manner and hence such materials exhibit perfect translational symmetry. However, many real materials, even though classified as crystals, have some imperfections and disorder present. On the other hand, on a large length scale the electrostatic potential in an amorphous sample is totally random due to the lack of long-range order in spatial coordinates of the nuclei. Even though classified as amorphous, over a short-range many real amorphous materials still retain order, which is similar to the corresponding crystalline materials^23^. All the real materials fall somewhere between the two extreme cases of being perfectly amorphous and being perfectly crystalline. In most materials the disorder arises from the imperfections caused by atomic vacancies, interstitial atoms, substitutional/impurity atoms, dislocated atoms etc. We make the assumption that, over a short-range, the amorphous PbO almost still retains the structure of the crystalline PbO with only one type of imperfection present on such scale. We test this hypothesis using DFT calculations. We make a number of plausible modifications to the experimental PbO crystal structure and then perform DFT calculations to evaluate the effects of these structural changes on the resulting spectra. The spectra calculated for these model modifications are compared to the measured spectra to determine the validity of such models for the short-range order in the amorphous sample. For such a model to be reasonable, it should reproduce the measured trends, which are the presence of the additional spectral weight below the main absorption feature in XAS and the shift of the CB edge to lower energy. In the XES for the amorphous sample, the model should produce additional DOS in such a way that the VB edge, unlike the CB edge, does not shift in energy. Note that we are not trying to model the amorphous sample on a large scale, which is currently computationally impossible. We are only trying to see if there is still short-range order, and if so then to first approximation what kind of imperfection is present at such length scale. We test only one type of imperfection at a time to keep it computationally feasible and see what effect it has on the x-ray spectra. We will not discuss energies of these different model configurations. It would make sense to discuss the energies of the configurations if we were making claims about the long-range order. We are arriving at our conclusion (which model can account for the additional spectral weight?) from the spectral shape only. We test the following four different short-range structural models:

#### Relaxed vs. unrelaxed(unperturbed) structures

**Figure 4:**
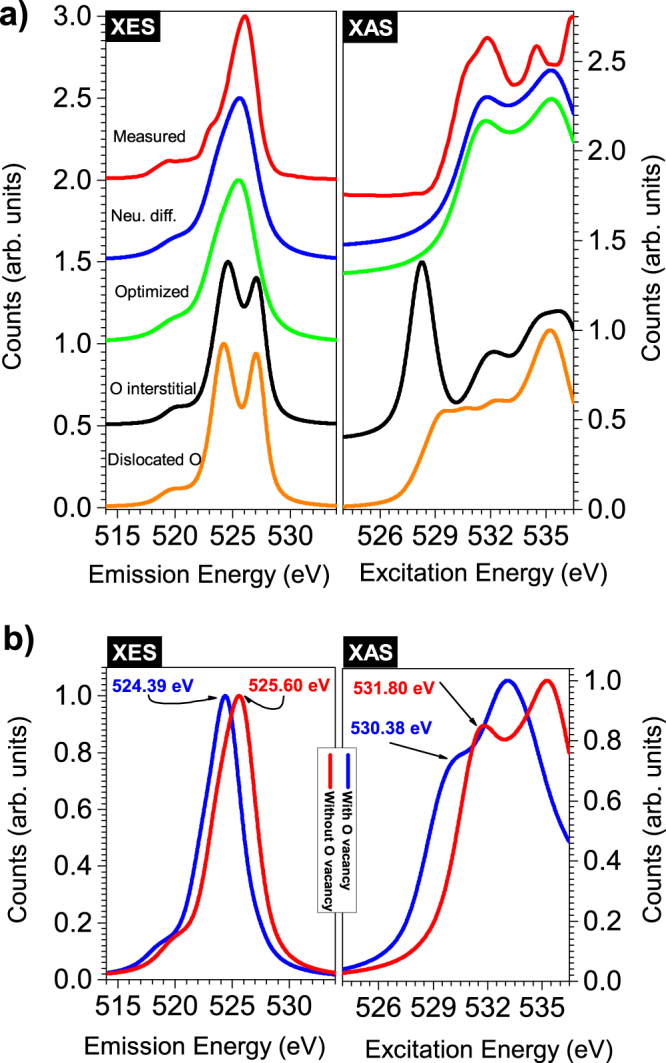
(**a**) Calculated O K edge spectra, emission on the left and absorption on the right, for the experimental (unperturbed) and for the three ruled out models. The red curves represent measured spectra for the amorphous PbO sample (**b**) Left panel: Calculated O K edge XES spectra for α-PbO with and without O vacancy. Right panel: Calculated O K edge XAS spectra. The experimental spectra for the amorphous sample are a weighted sum of the two spectra in each panel. The CB edge is determined by the blue curve in the left curve explaining why O vacancy lowers the CB edge for the amorphous samples. VB edge is determined by the red curve in the left figure which corresponds to α-PbO without O vacancy.

To determine whether the observed changes in DOS are due to differences in stress we perform a geometry optimization calculation on the α-PbO structure determined experimentally^24^. If there is significant stress in the experimentally determined structure, the crystal will not be in a state where the computed forces on the atoms are minimal (real forces must be zero). Therefore, a geometry-optimization, in which the forces are minimized, will lead to a different structure resembling the relaxed crystal. For the experimental structure, the computed net force on the Pb atom is −20.062533 mRy/Bohr along the c axis of the unit cell, while there is no net force on the O atom. This corresponds to a net force of −20.062533 mRy/Bohr per unit cell along the c axis. For the optimized structure, the forces are negligible on both Pb and O atoms and the coordinates for the O atom remain unchanged, while there is 2.5% decrease in the c coordinate of the Pb atom. The Pb atom coordinates change from (0, 0.25, 0.7655) to (0. 0.25, 0.7464) while O atom coordinates remain unchanged at (0.25, 0, 0). Please refer to the subsection ‘crystal structures’ for more details about the crystal structures. For a real crystal there must be a zero net force on each unit since it is in a state of equilibrium. If an external force is applied resulting in increased stress, the atomic/molecules positions will change in such a way that net force is again zero. In order to balance this force of 20.062533 mRy/Bohr there must be an additional force of the same magnitude, but in opposite direction which implies the presence of normal stress of 0.355080 mRy/Bohr^3^ along the c axis for the experimental structure. (this force can arise due to various imperfections in the real crystal). Our hypothesis is that on a small scale, the amorphous sample has regions resembling relaxed and regions resembling unrelaxed structure. If true, we should see a contribution from the both structures to the experimental spectra accounting for the additional DOS discussed above. We find that the differences between relaxed and experimental crystal structures produce negligible changes in the calculated x-ray spectra as shown in Fig. 4a. Hence, we rule out this model as a possible explanation for the observed additional DOS in the experimental spectra.

#### The O interstitial model

The O interstitial structure is modelled by introducing an extra O atom between two adjacent layers in the experimental structure of α-PbO, and subsequently minimizing the forces by performing a geometry optimization. Again our hypothesis is that on a small scale, the amorphous sample contains regions of unperturbed experimental structure, and regions of small perturbation due to O interstitial atoms. Calculated absorption and emission spectra for this model shown in Fig. 4a are very different than for the experimental (unperturbed) structure, and a linear combination of these two spectra can’t reproduce the measured spectra for the amorphous sample.

#### The O dislocation model

In this model we modify experimental structure by removing one of the O atoms from the layered structure of α-PbO and place it between two adjacent layers followed by a structural optimization calculation. This model again can’t explain the additional DOS observed because a linear combination of the spectra for perturbed and unperturbed structures can’t reproduce measured spectra for the amorphous sample. This model is also ruled out since the DFT calculations do not produce the spectra that follow the experimental trends.

#### The O vacancy model

**Figure 5:**
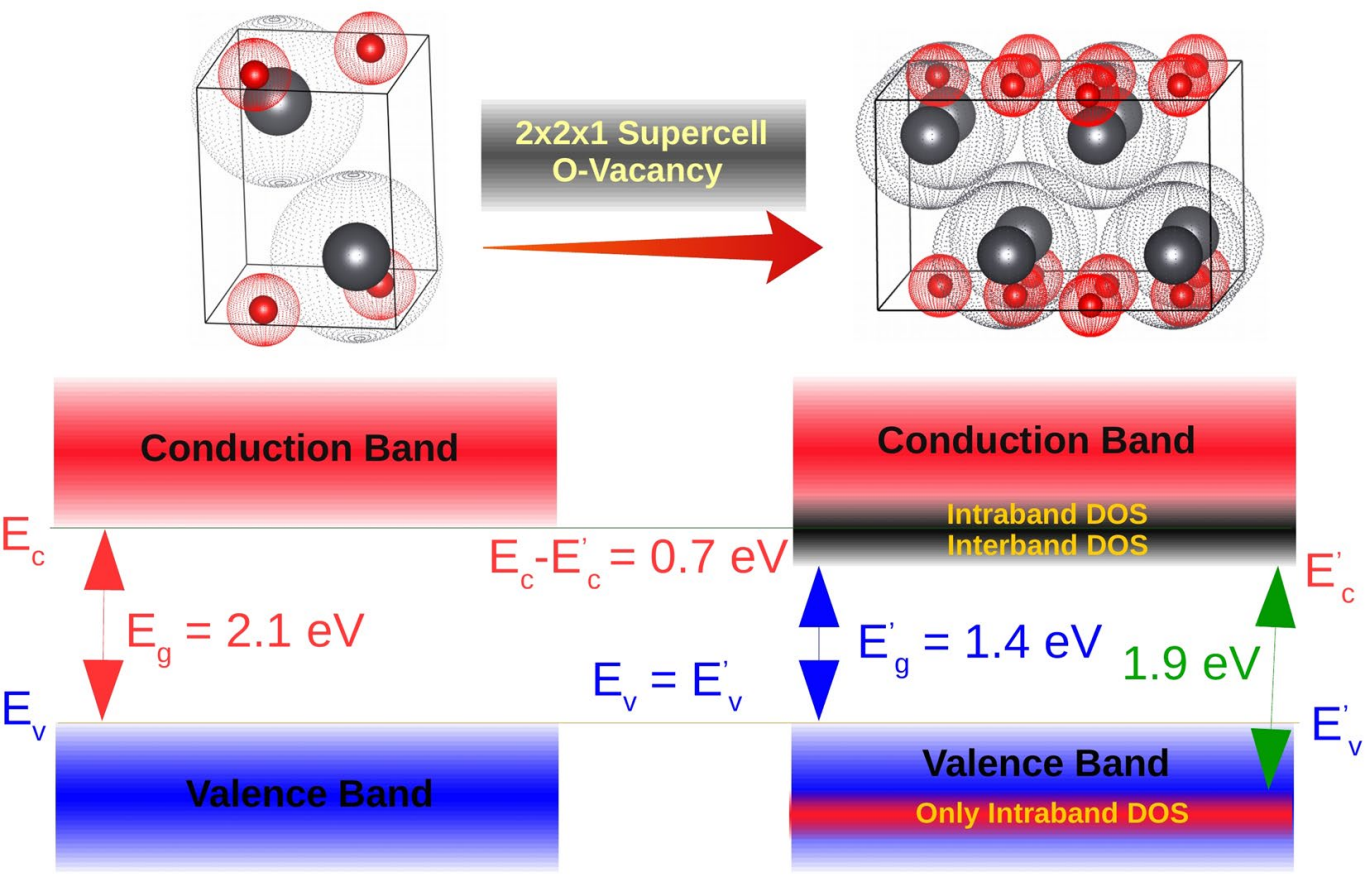
Graphical summary of the key findings – The left side of this figure shows experimental structure of α-PbO. The structure on the right is obtained by introducing an O vacancy and then performing geometry optimization calculations. DFT calculations incorporating an O vacancy reproduce the additional experimentally determined electronic states in all the amorphous samples. There is presence of additional inter- and intraband DOS in the CB (dark grey band), but only intraband DOS in the VB [small red band] for all the amorphous samples lowering their band gap value from to 1.4 eV.

A 2 × 2 × 1 supercell from experimentally determined α-PbO structure is constructed and one of the O atoms is removed from the supercell creating an O vacancy. Geometry optimization calculations are performed on this modified structure resulting in a structure shown on the right side in Fig. 5. Our hypothesis is that on a small scale, the amorphous sample contains regions of experimentally determined structure, and regions of O vacancy structure, but these structures do not represent the large scale random arrangements of the atoms for the amorphous samples. This is a promising candidate model and the calculation results for this model are discussed in detail below.

Our DFT calculations for α-PbO with an O vacancy agree very well with the measurements for both conduction and valence bands for the amorphous samples. The right side of Fig. 4b shows calculated O K edge XAS spectra for α-PbO with (O K edge for an atom near the O vacancy) and without O vacancy (the same atom without any vacancy). The first peak in XAS which appears at 531.80 eV for α-PbO shifts to 530.38 eV for α-PbO with O vacancy. This amounts to a shift of 1.42 eV. Figure 3 shows that the main peak in measured XAS for the amorphous sample appears at 531.80 eV with a shoulder at 530.40 eV (determined by peak deconvolution analysis). These two peaks are 1.40 eV apart. Since the measured spectra are a sum of the absorption from unaffected and affected sites near the O vacancy, both of these features appear in the experimental spectra for the amorphous samples. Thus with this model, we can explain the additional spectral weight in the conduction band that we measured and discussed above. Our confidence in this model is further strengthened by comparing the changes in valence band with experimental XES. When we resonantly excite the a-PbO-1 sample along the very bottom of the conduction band, the XES spectrum shows a usual expected peak at 525.57 eV and an additional peak at 524.28 eV (see left side of Fig. 3). These two measured peaks are 1.29 eV apart. The right hand side of Fig. 4b shows that the peak in emission for the α-PbO without O vacancy appear at 525.60 eV while for the structure with O vacancy this peak is shifted to the left by 1.21 eV and is located at 524.39 eV. Thus this model also explains the extra feature at 524.28 eV in the experimental RXES spectra for the amorphous samples. We conclude that PbO with an O vacancy is responsible for observed changes in valence and conduction band for the amorphous samples.

### Band gaps

Since XAS and XES probe the unoccupied and occupied partial density of states, respectively, it is relatively straight forward to display both spectra on a common energy axis and determined the band gap experimentally. The experimental values of the band gaps for the samples are determined using the second derivative method^25^. In this method, the band edges are determined by the first maxima (above the noise level) in the second derivative of the absorption and emission spectra (bottom panels in Fig. 1). This simple method has advantages over the more widely used linear extrapolation method because these values are more reproducible and less ambiguous (than values from linear extrapolation). We also have to estimate and correct for the shift in the CB edge due to the presence of an O 1 s core hole since XAS probes the CB in the presence of an O 1 s hole – not the ground state which is what band gap refers to. This is determined by comparing the calculated ground state XAS with the calculated excited state XAS. A 2 × 2 × 2 supercell is created and one 1 s electron from one of the O atoms is removed to simulate core hole effect. Since it is not feasible to run calculations using a large enough supercell, the core hole concentration is too high. As a result, this method overestimates the core hole effect, but this is not an issue here since for all the calculated spectra this correction is less than 0.05 eV, much smaller than the experimental uncertainty. The reported experimental band gap values for α-PbO and β-PbO are about 1.9 eV and 2.5 eV, respectively^1,17^. Our experimental data show the band gaps for both the α-PbO and β-PbO samples are 1.9 ± 0.2 eV. The β-PbO band gap is too small due to the α-PbO impurity. As for our PbO films, measured band gap values are 1.4 ± 0.2 eV for all three a-PbO samples and 2.1 ± 0.2 eV for the p-PbO sample. Therefore, the experimental value for p-PbO agrees well with α-PbO. However, for the a-PbO samples, the experimental band gap is 0.7 eV smaller than the p-PbO sample, and does not agree with the literature values for any of the PbO phases. This narrowing of the band gap is not surprising given the findings discussed above that the conduction band shifts to lower energy for the a-PbO due to the presence of additional states, while the valence band edge is unaffected. The PBE-GGA potential produces calculated band gap values of 1.3 eV for α-PbO and 2.1 eV for β-PbO, while the mBJ potential gives 1.7 eV for α-PbO and 2.3 eV for β-PbO. Although mBJ provides a better agreement with the experimental values, it still underestimates the band gap for both PbO phases. The mobility band gap for all a-PbO is measured to be 1.9 eV. This is again not surprising because the additional DOS in an amorphous material is expected to be localized and hence do not contribute to the current^21^.

## Conclusion/summary

This work provides strong experimental and theoretical evidence that the electronic structure of PbO photoconductors depend on the growth condition. An amorphous PbO layer deposited using the ion-assisted thermal evaporation technique, exhibits band tailing of about 0.7 eV at the bottom of the conduction band. This band tailing effect is not observed in polycrystalline PbO film deposited by basic thermal evaporation technique (Fig. 5). The band gap of the amorphous sample subsequently decreases by about 0.7 eV in comparison to the polycrystalline PbO sample. These findings are summarized in Fig. 5 which illustrates the presence of both interband and interaband additional DOS near the bottom of CB, but only intraband additional DOS in the VB for the amorphous PbO samples. As a result, only the CB edge exhibits band tailing. We also show that on a small scale, the amorphous sample does have some periodicity giving rise to a well-defined localized additional DOS. This narrowing of the band gap is likely due to the presence of O vacancies in the amorphous sample, even though on a larger scale the Pb to O ratio is 1:1. Thermal annealing can eliminate these additional states in the amorphous samples. The width of the band tail below the conduction band is larger than that in a-Se – the only amorphous photoconductor employed today in direct conversion imaging detectors. Therefore, technology optimization must target reducing the density of states (DOS) in the vicinity of the conduction band that in turn requires further study the electronic structure to provide a better insight on the exact DOS distribution.

## Methods and Materials

### Crystal structures

Two of the most common phases of PbO are the layered alpha and beta phases. α-PbO has a tetragonal space group (*P*4/*nmm*) with unit-cell parameters: a = 5.6253 Å, b = 5.6253 Å, c = 5.0259 Å, α = β = γ = 90° which are determined using neutron diffraction at 300 K^24^. The atomic nuclei coordinates are: Pb: (0, 0.25, 0.7655), O: (0.25, 0, 0). β-PbO has an orthorhombic space group (*Pbcm*) with unit-cell parameters: a = 5.8931 Å, b = 5.4904 Å, c = 4.7528 Å, α = β = γ = 90°. These experimental unit cell parameters are also determined by neutron diffraction at 295 K^26^. The atomic nuclei coordinates are: Pb: (0.2297, −0.0116, 0.25), O: (−0.1347, 0.0917, 0.25).

### Samples

The seven differently prepared lead oxide samples include polycrystalline PbO (p-PbO) deposited on ITO covered glass substrate, two amorphous PbO deposited on ITO covered glass substrate (a-PbO-1, a-PbO-2), one amorphous PbO deposited on Aluminum substrate (a-PbO-3) powdered α-PbO, and β-PbO are studied. One sample is prepared by thermally annealing a-PbO-1, which transforms it to a polycrystalline (a-PbO-1 annealed). The 7 μm thick poly-PbO sample is prepared by the basic thermal vacuum evaporation technique. In this technique, high purity (5 N) PbO powder is evaporated at ~1000 °C in the atmosphere of molecular oxygen, directly supplied to the chamber. Evaporated PbO particles condense on the 2.5 × 2.5 cm^2^ ITO covered glass substrate. The grown poly-PbO layer consists of a porous network of randomly oriented platelets ~1 × 1 × 0.1 micron in size. The platelets inside are mainly composed of the alpha (tetragonal) phase of PbO, although grown layers start with a thin seeding layer of beta (orthorhombic) phases of PbO^1,27,28^. The a-PbO layers are deposited with the ion assisted thermal evaporation technique where the major difference from the p-PbO deposition process is that molecular oxygen is supplied to the chamber through the commercial ion source, ionizing the oxygen and accelerating the ions. This advancement of the deposition process triggers a phase change of the growing layer from polycrystalline to amorphous^12^. The a-PbO-1 sample is thermally annealed at 400 °C for one hour. In order to prevent any chemical reaction between PbO and ambient gasses, annealing takes place under the protective atmosphere of Ar gas.

### Measurements

The count rates in both x-ray absorption and emission spectra are proportional to the square of the scattering matrix element between the initial and the final states multiplied by the density of states. In absorption spectroscopy, the transition matrix elements are vanishing for transitions from 1 s to occupied states due to the Pauli-exclusion principle, and are appreciable only for those transitions where ∆l = ±1, which is referred to as dipole selection rule. Hence, the O K-edge XAS probes the partial density of unoccupied p-states in the conduction band. Similarly, the O K-edge XES probes the partial density of occupied p-states in the valence band.

Three different beamlines, REIXS^29^ and SGM^30^ at CLS, and BL 8.0.1^31^ at ALS are used for our measurements. Resolving power, E/∆E, values for the monochromator at the SGM, REIXS, and BL 8.0.1 are 5000, 8000, and 7000, respectively. These correspond to an energy resolution value ∆E of 0.1 eV, 0.06 eV and 0.07 eV at 500 eV for XAS respectively. E/∆E values for the spectrometer at the REIXS and BL 8.0.1 are 2000 and 800, respectively, which translate to an energy resolution of 0.25 eV and 0.6 eV at 500 eV for XES respectively. All spectra are collected at the O K-edge at room temperature with a sample to detector angle of 45°. All of the spectra are calibrated by measuring O K-edge XAS and XES spectra for bismuth germanium oxide (BGO) and comparing their features to the known energy values (517.9 eV and 526.0 eV for XES, and 532.7 eV and 538.6 eV for XAS).

### DFT calculations

The Wien2K package software is used to perform calculations in the framework of density functional theory^32^. Experimentally determined crystal structures for pure phases of α-PbO, and β-PbO, discussed above, are used as input^24,26^. For all the crystals, the PBE-GGA exchange correlation potential is employed. The energy value to separate core and valence states used is −6 Ry. A value of 7.00 is used for R_MT_K_MAX_. R_MT_ values are set by using almost touching atomic radii spheres. The following convergence criteria are used for ground state calculations: E = 0.0001 Ry, F = 1 mRy/au, Charge = 0.001 e. For α-PbO and β-PbO, the k-mesh inputs of 26 × 26 × 20 and 20 × 22 × 25 are used, respectively. The’final state rule’ states that the x-ray spectra depend on the final quantum state. For the K edge absorption, the final state has a core hole in the 1 s level. To incorporate this effect, a supercell of 2 × 2 × 2 is created for all three crystals, one of the core electrons (1 s) is removed from one of the oxygen atoms in the supercell and is added to the background lattice charge. GGA calculations are known to underestimate band gaps. A modified version of the exchange potential (mBJ) proposed by Becke and Johnson, which takes PBE-GGA calculation results as input, is used to calculate the band gap values^33^.

### Data availability

Raw and processed data generated and analyzed during this study are available from the corresponding author on reasonable request.
